# A Case of Unilateral Spatial Neglect and Impaired Dorsal Attention Network Function Due to Acute Subcortical Hemorrhage: Focusing on Endogenous Attention While Walking

**DOI:** 10.7759/cureus.98113

**Published:** 2025-11-29

**Authors:** Atsushi Iwasa, Takato Nishida, Hideki Nakano

**Affiliations:** 1 Department of Rehabilitation, Ookuma Hospital, Nagoya, JPN; 2 Department of Physical Therapy, Faculty of Health Sciences, Kyoto Tachibana University, Kyoto, JPN; 3 Faculty of Rehabilitation and Care, Seijoh University, Tokai, JPN; 4 Graduate School of Health Sciences, Kyoto Tachibana University, Kyoto, JPN

**Keywords:** dorsal attention network, stroke, unilateral spatial neglect, visuospatial attention network, walking

## Abstract

Unilateral spatial neglect is a higher-level cognitive disorder characterized by difficulty perceiving or responding to stimuli in the contralateral space due to lesions in the cerebral hemisphere. Presently, there are clinical challenges in the assessment of and intervention for unilateral spatial neglect. First, assessments and interventions are often conducted under static conditions, such as sitting, which can lead to discrepancies in the symptoms observed during daily activities, such as walking. Second, although unilateral spatial neglect affects both the peripersonal and extrapersonal space domains, conventional interventions have mainly targeted the peripersonal space domain, with insufficient attention to the extrapersonal space domain. In this case report, we aimed to present a case in which we evaluated walking conditions, using a treadmill, and an endogenous attention task targeting the extrapersonal space domain, using a laser pointer, in a 50-year-old male with a hemorrhage in the right superior parietal lobe. The evaluation revealed discrepancies between seated and walking conditions. Following the intervention, the participant’s attention span toward the left side improved during walking, and walking ability increased after 5 days. The intervention continued, and the participant was discharged 1 month after the onset of cerebral hemorrhage. This suggests that evaluations and interventions targeting extrapersonal spaces under dynamic conditions that are more closely aligned with daily life may be effective for symptom improvement, addressing issues that cannot be fully captured by desk-based tests alone.

## Introduction

Unilateral spatial neglect (USN) is a higher-level brain dysfunction characterized by difficulty in noticing or responding to stimuli in the contralateral space due to lesions in the cerebral hemisphere. Involvement of the visuospatial attention network in the pathophysiology has been suggested, and an understanding of its function is crucial for effective rehabilitation [1]. The visuospatial attention network is broadly divided into two categories: the dorsal attention network (DAN), associated with goal-directed attention, and the ventral attention network (VAN), associated with stimulus-driven attention. The DAN connects to the superior parietal lobule, superior frontal gyrus, and anterior cingulate cortex and is associated with top-down endogenous attention. The VAN is connected to the temporoparietal junction and middle and inferior frontal gyri, and it is associated with bottom-up exogenous attention [2]. In clinical settings, there is often a discrepancy between the results of desk-based tests and the symptoms observed in daily living activities such as walking. Therefore, it is necessary to evaluate and intervene in the visuospatial attention network under dynamic conditions closer to daily living activities.

Presently, the Behavioral Inattention Test (BIT) is widely used to assess USN, and it is useful for evaluating endogenous attention, which is controlled by the DAN. However, the BIT is generally evaluated in seated conditions [3]. Additionally, the Catherine Bergego Scale (CBS) is commonly used, and its usefulness in detecting USN in daily life relative to traditional desk-based tests has been observed [4-6]. However, this approach does not clearly distinguish between endogenous and exogenous attention. Besides, there are reports of the use of the Modified Posner Task [6-8]. However, none of these evaluations have been conducted under dynamic conditions, such as walking. Thus, evaluation methods generally used in clinical practice involve static conditions at a desk, and evaluations that consider endogenous attention under dynamic conditions, such as walking, are insufficient. Therefore, even patients who have a clear cut-off value and are not diagnosed with USN may exhibit neglect symptoms during daily living activities.

Regarding intervention methods, approaches such as prism adaptation, visual search training, and trunk rotation have been reported for endogenous attention; however, these interventions are performed in seated conditions and do not involve dynamic scenarios such as walking conditions [9-11]. Furthermore, the spatial domain in which USN occurs can be divided into peripersonal and extrapersonal spaces [12]. Spaccavento et al. reported that visual search training and prism adaptation did not improve the extrapersonal space, although they improved the peripersonal space [13]. Therefore, it is necessary to consider approaches targeting not only the peripersonal space but also the extrapersonal space, in addition to traditional approaches focused on the peripersonal space. Therefore, in this study, we aimed to present a case in which we evaluated cases that showed a decline in endogenous attention, which is a part of the visuospatial attention network, using a treadmill walking condition and endogenous attention tasks targeting the peripersonal and extrapersonal spaces.

In this case report, we aimed to present the case of a patient who showed a decline in endogenous attention, which is a part of the visuospatial attention network, using a treadmill walking condition and endogenous attention tasks targeting the peripersonal and extrapersonal spaces.

## Case presentation

Participant characteristics

**Figure 1:**
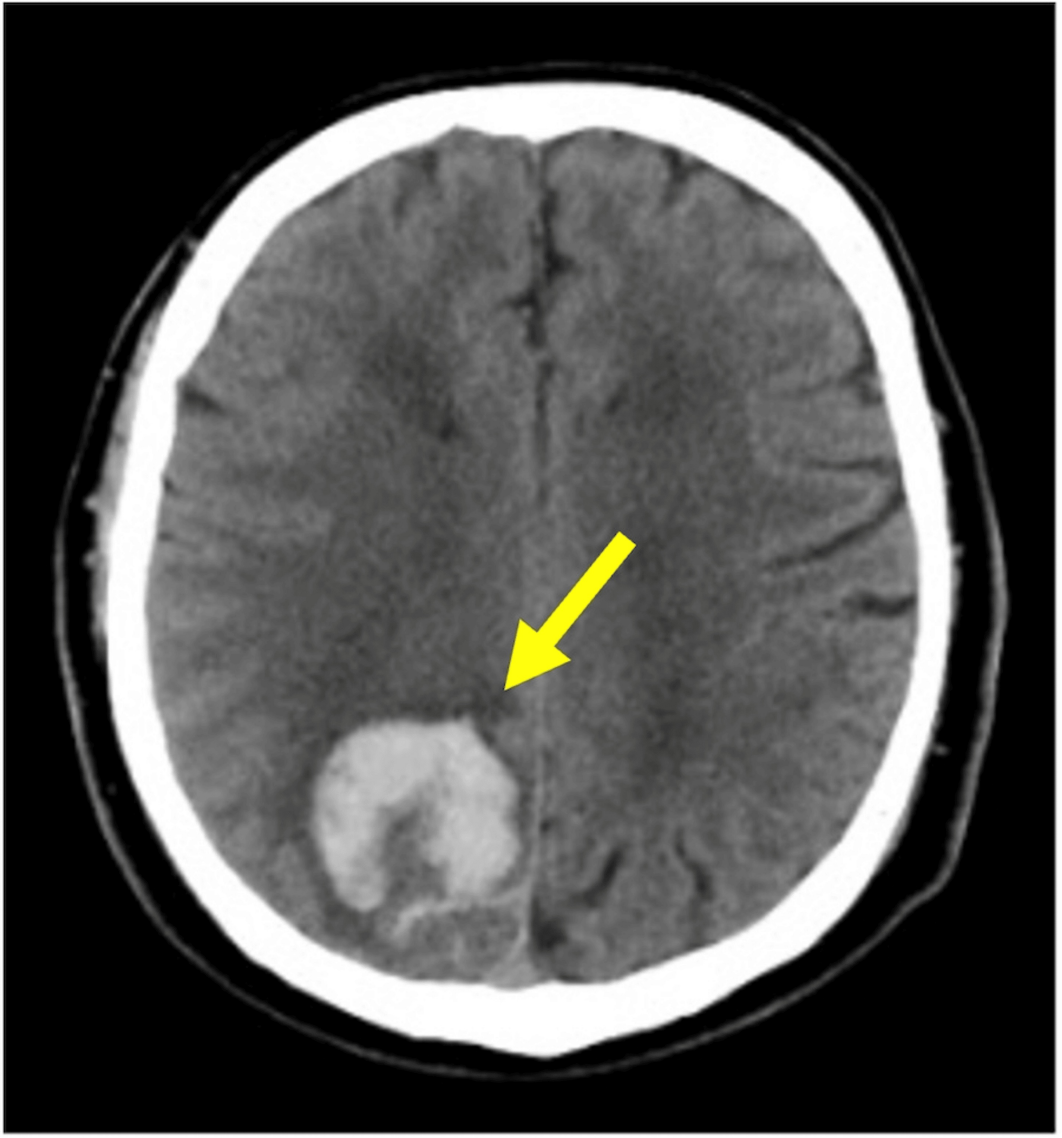
Brain image of the study patient. Computed tomography (CT) scan on day one post-stroke. The yellow arrow indicates the acute subcortical hemorrhage in the right superior parietal lobe.

The patient was a 50-year-old right-handed male diagnosed with a subcortical hemorrhage. Before symptom onset, the patient was able to walk independently and was employed. The patient’s present medical history included discomfort in the head and confusion about how to get to a frequently visited location for work, leading to a call for emergency assistance by the patient’s colleagues and hospitalization on the same day. At admission, computed tomography revealed hemorrhage in the right superior parietal lobe (Figure 1). The National Institutes of Health Stroke Scale score was 2 points, and the only points added were extinction and attention disorder. Physical therapy was initiated on the second day of hospitalization. Walking was initiated on the third day; however, the participant exhibited USN symptoms such as overlooking the entrance on the left side and turning right at the corners.

Evaluation of unilateral spatial neglect (USN)

**Figure 2:**
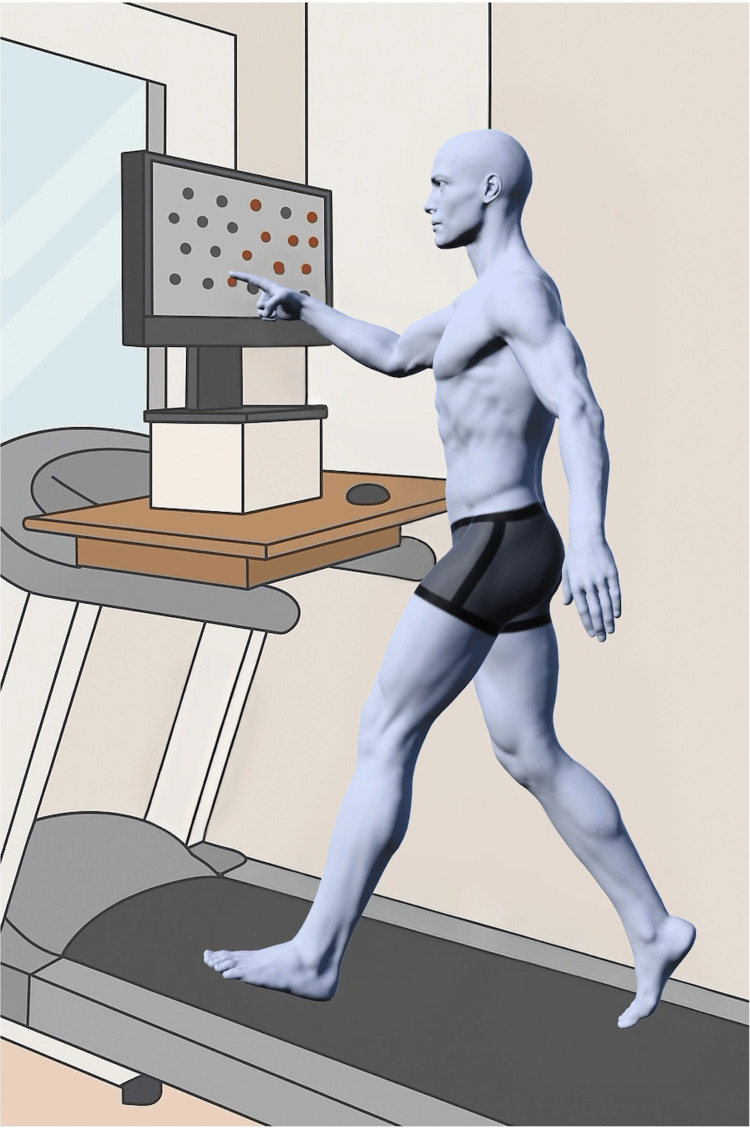
Methods for the assessment of endogenous and exogenous attention tasks during walking. The monitor was placed on the treadmill at eye level for the patient. The distance between the monitor and the patient was within the patient’s reach. The walking speed was set to a comfortable speed for the patient. This figure was created by the authors for this case report.

The BIT, which has a wide clinical application, was used to evaluate USN, and the CBS was used to evaluate behavior in daily life. The BIT is a copyrighted instrument. Its use in this case was covered by an institutional license held by Ookuma Hospital. Endogenous and exogenous attention were evaluated using an evaluation device (@ATTENTION, Creact Corp., Tokyo, Japan) equipped with a touch panel personal computer under seated and walking conditions [2]. The @ATTENTION cognitive assessment and rehabilitation system is a commercial product. In the present case, the equipment was used with the express permission of Professor Takehiko Yamanaka of Nihon Fukushi University, who provided access to the device. In the seated condition, the participant sat on a chair with a monitor placed on a table in front of them. The monitor was positioned at eye level and within reach of the arm. In the walking condition, the monitor was placed on a treadmill (Senoh Corp., Chiba, Japan) at eye level and within arm’s reach (Figure 2). Walking speed was determined based on the results of a 10-m walking test and set to a safe walking speed on the treadmill [14-15]. Additionally, walking practice for a few minutes was conducted to ensure safe treadmill walking before proceeding.

**Figure 3:**
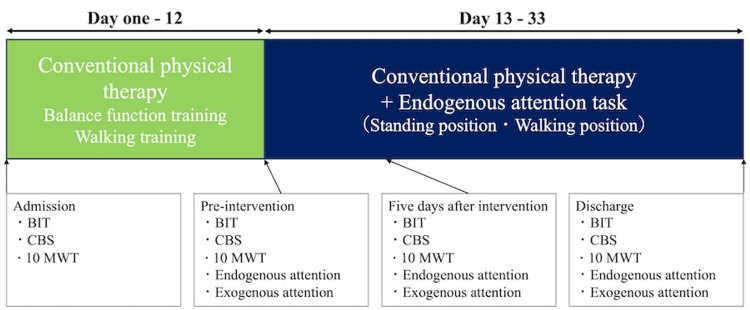
Conventional physical therapy and intervention timeline and assessment. BIT: Behavioral Inattention Test [3]; CBS: Catherine Bergego Scale [4,5]; 10 MWT: 10-m walking test [14].

Endogenous attention was assessed using an endogenous attention task in which the participant was instructed to select objects using their index finger. The participant was instructed to select all objects in any order, and the numbers of selected objects, unselected objects, and objects selected multiple times were evaluated. Exogenous attention was assessed using an exogenous attention task. Further, the participant was instructed to select objects that flashed randomly with a 5-s timeout, and the spatial distribution of reaction times was calculated as a left-to-right ratio [2]. The evaluation periods were admission, before the intervention, 5 days after the intervention, and discharge (Figure 3).

Intervention

On the second day of hospitalization, conventional physical therapy focusing on balance and walking was started. On the 13th day, a treadmill was introduced to aid endogenous attention in addition to conventional physical therapy. Endogenous attention tasks were used for the intervention because brain imaging revealed lesions in the superior parietal lobule (Figure 1), suggesting DAN dysfunction. Therefore, an endogenous attention task was selected to restore DAN function.

**Figure 4:**
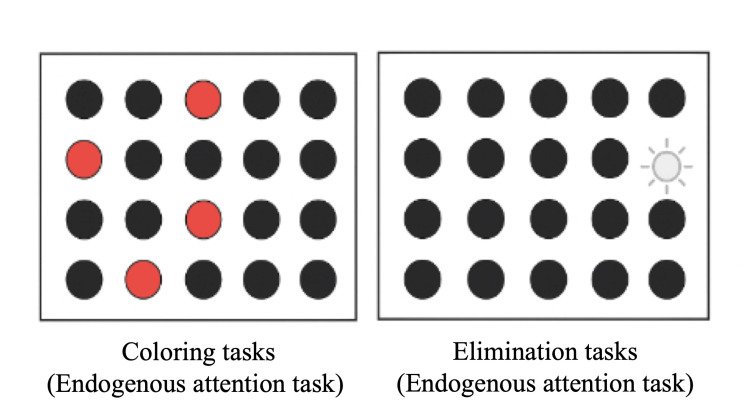
Rehabilitation tasks for peripersonal space. The left panel shows a coloring task in which the selected object changes from black to red, and the right panel shows an elimination task in which the selected object disappears from the screen. This figure was created by the authors for this case report.

**Figure 5:**
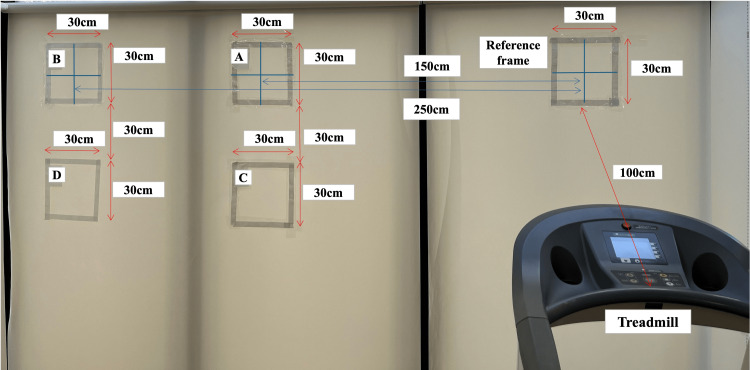
Rehabilitation tasks for extrapersonal space (endogenous attention task). The figure demonstrates an endogenous attention task using a laser pointer in extrapersonal space. A reference frame with sides measuring 30 cm was placed 100 cm from the treadmill. Frame A of the same size was positioned 150 cm to the left of its center, and frame B was positioned 250 cm to the left. Additionally, frames C and D, each of the same size, were placed 30 cm below frames A and B, respectively. The therapist used a laser pointer to indicate points on each frame from behind the patient, and the patient explored the indicated points. (1) The patient walks on the treadmill and gazes at the reference frame in front of them. (2) The therapist randomly presents a point with a laser pointer in any of frames A–D. (3) After the therapist gives verbal instructions, the patient explores the presented points and verbally answers the letter in the box where the points are presented. (4) After exploration, the patient returns gaze to the reference frame, and the therapist randomly presents a point in each of frames A–D once. A total of four points were presented as one set, and steps (1) to (4) were repeated for 10 min. This figure was created by the authors for this case report.

Regarding the peripersonal space, we used the @ATTENTION rehabilitation tool and performed coloring and elimination tasks (Figure 4) for 5 min each. Figure 5 shows the design of the extrapersonal space. The positions of frames A to D were determined after the therapist confirmed that they were not visible when focusing on the reference frame to avoid creating an exogenous attention task. The therapist indicated each frame with a laser pointer behind the participant, and the participant searched for the indicated point. The specific procedure was as follows: (1) the participant walked on a treadmill and gazed at the reference frame in front of them; (2) the therapist randomly presented a point with a laser pointer in any of frames A-D; (3) after the therapist provided verbal instructions, the participant explored the presented points and verbally answered the letter in the box where the points were presented; (4) after the exploration, the participant returned gaze to the reference frame, and the therapist randomly presented a point in each of frames A-D once. Four points were presented as one set, and steps (1)-(4) were repeated for 10 min.

Progress

At the time of admission, the Fugl-Meyer assessment showed that the upper limbs had 66 points and the lower limbs had 34 points, indicating no motor paralysis of the left upper and lower limbs [16]. The Mini-Mental State Examination (MMSE) score was 23 points, indicating no symptoms that would interfere with the evaluation or intervention [17]. The MMSE is a copyrighted instrument. Its use in this case was covered by an institutional license held by Kyoto Tachibana University. Additionally, no visual field defects such as hemianopia were observed. The results of the USN evaluation showed that the patient scored 54/146 points on the BIT, and all items were below the cut-off value. The CBS scores were 1 for participant self-assessment total score and 11 for the objective total score (Table 1); no symptoms of neglect of personal space were observed, but symptoms of neglect of peripersonal and extrapersonal spaces were observed. The Functional Ambulation Categories (FAC) score was 3, the 10-m walking speed was 0.53 m/s (Table 2), and the participant was able to walk without assistance in the hospital [18]. However, the patient overlooked entrances and exits on the left, turned right around corners, and became lost, indicating the need for supervision.

**Table 1:** Serial assessment of spatial attention. Endogenous attention numbers indicate the number of objects that can be selected. The left-right ratio indicates the difference in reaction time between the left and right spaces. A value close to 1.0 indicates that there is no difference in reaction time between the left and right spaces. BIT: Behavioral Inattention Test [3]; CBS: Catherine Bergego Scale [4,5]

		Admission	Pre-intervention	5 Days after Intervention	Discharge
BIT	Line crossing	16	18	36	36
	Letter cancellation	19	21	26	38
	Star cancellation	18	31	24	54
	Figure and shape copying	0	1	0	4
	Line bisection	0	0	0	7
	Representation drawing	1	0	0	1
	Total score	54	71	86	140
CBS (Objective)	Grooming	0	0	0	0
	Dressing	0	0	0	0
	Eating	1	0	0	0
	Mouth cleaning	0	0	0	0
	Gaze orientation	2	2	1	0
	Left limb knowledge	0	0	0	0
	Auditory attention	2	2	1	0
	Collisions	1	1	0	0
	Spatial orientation	3	3	1	1
	Finding personal belongings	2	1	0	0
	Total score	11	9	3	1
CBS (Patient self-assessment)	Total score	1	3	0	0
Endogenous attention	Sitting condition	-	15	25	35
	Walking condition	-	10	20	35
Exogenous attention (Left/Right ratio)	Sitting condition	-	1.50	1.20	1.13
	Walking condition	-	1.61	1.24	1.17

**Table 2:** Results of gait assessment. Progression of the 10 MWT and FAC. 10 MWT: 10-m walking test [14]. FAC: Functional Ambulation Categories [18]

	Admission	Pre-intervention	5 Days after Intervention	Discharge
10 MWT (m/s)	0.53	0.53	1.11	1.22
FAC	3	3	4	4

**Figure 6:**
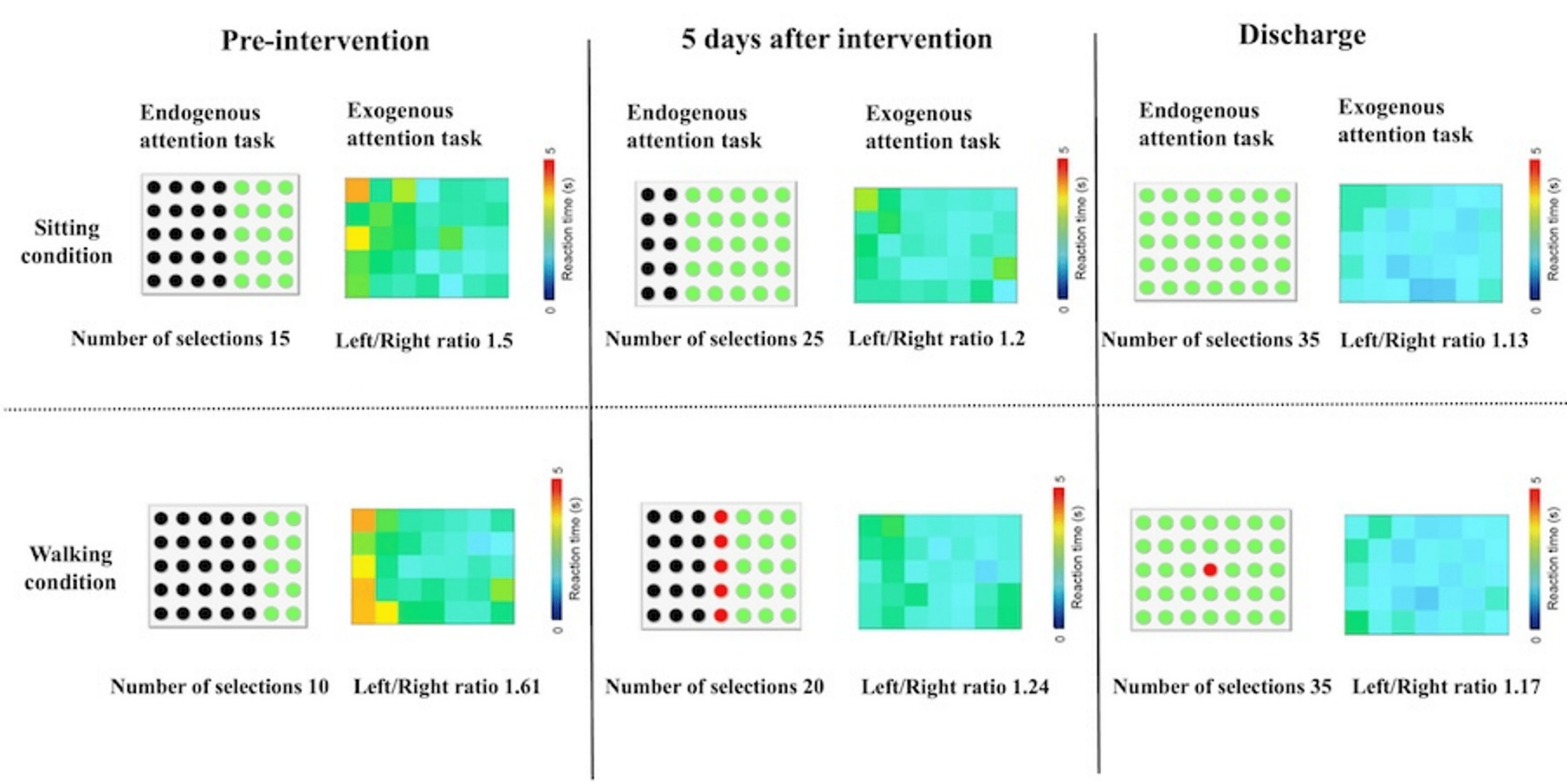
Results of the endogenous and exogenous attention tasks. The upper row shows the results for the sitting condition, and the lower row shows the results for the walking condition. The endogenous attention task: green indicates a location that was selected, black indicates a location that was omitted, and red indicates a location that was selected more than once. The number of selections indicates the number of objects that could be selected. The reaction time of the exogenous attention task is indicated in colors according to the number of seconds required for the selection. Red indicates the highest value (5 s), and the closer to blue (0 s), the lower the value. The left-right ratio is calculated by dividing the average reaction time of the 15 left objects by the average reaction time of the 15 right objects; a value greater than 1.0 indicates a slow response on the left side and may reflect neglect on the left side. This figure was created by the authors for this case report.

The pre-intervention assessment revealed a BIT score of 71/146 points and a CBS score of 3 points for the participant’s self-assessment total score and 9 points for the objective total score, showing minimal changes compared with the time of admission (Table 1). Walking ability, as measured using a 10-m walking speed of 0.53 m/s and FAC score of 3 (Table 2), showed no changes compared with the time of admission, and monitoring was required. Endogenous attention showed omissions in the four left columns under seated conditions and the five left columns under walking conditions, indicating a greater decline in endogenous attention in the left spatial area under walking conditions. Exogenous attention showed a reaction time left-right ratio of 1.5 under seated conditions and 1.61 under walking conditions, indicating delayed reactions in the left spatial area (Figure 6).

On the 13th day of hospitalization, an intervention using a treadmill was initiated in the peripersonal and extrapersonal spaces. At the start of the intervention, the patient required more time to explore objects on the left side than on the right side in the peripersonal and extrapersonal spaces.

After 5 days of intervention, the BIT score was 86/146, and the CBS score was 0 points for the participant’s self-assessment total score and 3 points for the objective total score (Table 1), indicating improvement. The FAC score was 4, and the 10-m walking speed was 1.11 m/s (Table 2), showing significant improvement and enabling independent walking in the ward. Regarding endogenous attention, the participant omitted the two left columns in the seated condition and the three left columns in the walking condition. However, compared with before the intervention, an expansion in the exploration range of the left space was observed. Regarding exogenous attention, the reaction time left-right ratio was 1.2 in the seated condition and 1.24 in the walking condition, indicating an improvement (Figure 6). However, during outdoor walking, the participant walked on the right side of the crosswalk and sometimes missed the hospital entrance when distracted.

On the 27th day of hospitalization, the patient was able to explore the left side without delay in an endogenous attention task using a laser pointer to focus on the extrapersonal space. On the 30th day of hospitalization, the patient was able to walk on familiar outdoor routes without getting lost. At the time of discharge, the FAC was 4, and the 10-m walking speed was 1.22 m/s (Table 2). We observed that the participant’s attention was generally directed to the left, even when walking outdoors. The BIT score was 140/146, exceeding the cut-off value, and the CBS improved to 0 points for the participant self-assessment total score and 1 point for the objective total score (Table 1). Endogenous attention showed no omissions on the left side in either the sitting or walking condition (Figure 6), and the participant was discharged on the 33rd day of hospitalization.

## Discussion

This case report discusses the visuospatial attention network in a case of acute subcortical hemorrhage presenting with left USN under seated (static) and walking (dynamic) conditions, with implementation of interventions for peripersonal and extrapersonal spaces under walking conditions.

The evaluation results revealed a decrease in endogenous attention. According to Corbetta and Shulman, the DAN is mainly responsible for endogenous attention, and it is connected to the superior parietal lobule, superior frontal gyrus, and anterior cingulate cortex [1]. In addition, Shomstein et al. reported that damage to the superior parietal lobule led to impaired endogenous attention [19]. In this case, a hemorrhage was observed in the superior parietal lobule on CT, suggesting that damage to the DAN may have contributed to a decline in endogenous attention.

In addition, discrepancies were observed between seated and walking conditions. Specifically, the number of objects omitted by the participant during the endogenous attention task increased from four left columns in the seated condition to five left columns in the walking condition. This suggests that the range of endogenous attention to the left space was further narrowed under walking conditions closer to real-life situations. Moreover, this result supports the clinical issue observed in practice, where symptoms in daily life activities, such as walking, do not align with the results of desk-based tests. Conventional, widely used tests such as the BIT and CBS cannot detect such changes in attention under dynamic conditions. Therefore, this report is significant, as it objectively demonstrates the discrepancy between clinical observations in desk-based tests and real-life conditions using concrete indicators and suggests the importance of evaluating dynamic conditions in USN cases.

In addition, we initiated an endogenous attention task for the peripersonal and extrapersonal spaces during walking. After 5 days of intervention, the CBS score improved from 9 to 3 points in the objective total score assessment; the result of the 10-m walk test improved from 0.53 m/s to 1.11 m/s, the FAC score increased from 3 to 4, and endogenous attention showed an expanded search range in left space. In the hospital ward, the patient no longer missed the left space or collided with objects on the left side of the body; additionally, the patient achieved independent walking. At the time of discharge, the participant had a BIT score of 140 points and an objective total score of 1 for the CBS, no longer missed the left space in endogenous attention in walking conditions, and was generally able to explore the left space in outdoor walking, leading to independence in a familiar environment.

Shin et al. reported that visual search training significantly improved line bisection and star cancellation tasks and reduced reaction times in the left spatial field [20]. The recovery of USN is related to the restructuring of the neural network [21-22]. In the present case, the endogenous exploratory task in the early stage of USN recovery may have promoted neuroplasticity and contributed to the restructuring of the neural network. In addition, the present study is unique in that it was conducted under a double-task condition, combining an endogenous attention task during walking, as opposed to conventional static conditions such as sitting. Such dynamic interventions in situations such as daily activities may promote the restructuring of the neural network. Therefore, in the rehabilitation for USN, it is important to incorporate early interventions under dynamic conditions in addition to those under static conditions.

This case report has some limitations. Owing to the single case, a similar recovery may not be achieved in other patients with USN. Additionally, a larger randomized controlled trial is required to clarify the causal relationship between spontaneous recovery and intervention during the acute phase of the disease. Furthermore, brain imaging is required to analyze the mechanism of recovery in detail.

## Conclusions

We evaluated a participant with USN due to an acute subcortical hemorrhage and mainly impaired endogenous attention during walking, and found a discrepancy between the results in the sitting and walking conditions. In addition, due to the performance of endogenous attention tasks in the peripersonal and extrapersonal spaces under walking conditions, the BIT, CBS, endogenous attention, FAC, and 10-m walk speed improved, indicating an improvement in walking ability. We believe that it is important to conduct assessments that consider the visuospatial attention network under dynamic conditions, such as walking, from an early stage, and to intervene after understanding the pathophysiology.
